# Size-Dependent Emission Enhancement in Deep-Ultraviolet AlGaN Microrods

**DOI:** 10.3390/nano16060355

**Published:** 2026-03-14

**Authors:** Xu Sun, Ziwen Yan, Tong Xu, Jiajun Zhu, Zili Xie, Xiangqian Xiu, Dunjun Chen, Bin Liu, Yi Shi, Rong Zhang, Youdou Zheng, Peng Chen

**Affiliations:** Jiangsu Provincial Key Laboratory of Advanced Photonic and Electronic Materials, State Key Laboratory of Spintronics, School of Electronic Science and Engineering, Nanjing University, Nanjing 210093, China; 502022230048@smail.nju.edu.cn (X.S.); dg21230063@smail.nju.edu.cn (Z.Y.); xt@smail.nju.edu.cn (T.X.); 502024230059@smail.nju.edu.cn (J.Z.); xzl@nju.edu.cn (Z.X.); xqxiu@nju.edu.cn (X.X.); djchen@nju.edu.cn (D.C.); bliu@nju.edu.cn (B.L.); yshi@nju.edu.cn (Y.S.); rzhang@nju.edu.cn (R.Z.); ydzheng@nju.edu.cn (Y.Z.)

**Keywords:** AlGaN microrods, deep-ultraviolet emission, cathodoluminescence, size effect

## Abstract

High-Al-content AlGaN microrods represent an effective platform for engineering deep-ultraviolet (DUV) emission. Here, we fabricated AlGaN microrods with varying diameters (2, 3, and 4 μm) via a top-down approach involving inductively coupled plasma dry etching followed by a KOH wet chemical modification. Their crystallographic facets and size-dependent optical properties were systematically investigated using scanning electron microscopy (SEM), cathodoluminescence (CL) spectroscopy, and CL mapping. We found that the KOH treatment selectively forms a-plane-dominated sidewalls on the high-Al-content portion of the microrods, whereas the etch pit bottoms stabilize as m-plane facets. Notably, the CL spectra show that the band-edge emission intensity of the 2 μm microrods is enhanced by a factor of 3.76 compared to the 4 μm structures. CL mapping further unveils the competitive dynamics between radiative recombination within the quantum wells and non-radiative recombination at surface states. These findings pinpoint 2 μm as the optimal diameter among the investigated range for maximizing spontaneous emission from these high-Al-content AlGaN microrods.

## 1. Introduction

AlGaN-based materials are pivotal for the development of deep-ultraviolet (DUV) optoelectronic devices for applications including sterilization, biochemical sensing, optical communication, and high-resolution lithography [1,2,3,4]. This is attributed to their direct and tunable wide bandgap, which covers the UVC spectral region. Nevertheless, the external quantum efficiency (EQE) of AlGaN-based DUV light-emitting devices remains disappointingly low compared to that of InGaN-based visible emitters, especially for wavelengths below 250 nm [5], which severely limits their practical applications.

Micro- and nanostructured architectures have emerged as a promising strategy to address these efficiency bottlenecks in III-nitride optoelectronics. Microrod structures, in particular, are advantageous due to their ability to facilitate strain relaxation, enhance light extraction, and control optical modes. The inherent large surface-to-volume ratio of microrods allows for effective strain relief, thereby suppressing the quantum-confined Stark effect (QCSE) [6]. Moreover, these structures function as optical microcavities, which can tailor the local photonic density of states and modulate spontaneous emission via the Purcell effect, ultimately improving the radiative recombination efficiency.

In the GaN-based material system, microrod and microdisk structures have demonstrated low-threshold lasing and effective confinement of whispering-gallery modes (WGMs) [7,8]. To understand and optimize these optical properties, it is essential to resolve the underlying carrier recombination mechanisms at the sub-micron scale. Cathodoluminescence (CL) spectroscopy has proven to be an indispensable tool for probing these micro-architectures, as it offers a superior spatial resolution to resolve carrier recombination at the nanoscale. For instance, CL has been successfully employed to investigate the facet-dependent emission and defect distribution in GaN-based nanostructures [9], as well as the intricate carrier dynamics and surface recombination velocities in III-nitride nanowires [10], Furthermore, advanced CL studies have elucidated how structural inhomogeneities and point defects, such as carbon impurities, influence the overall radiative efficiency [11,12]. Beyond axial structures, radial heterostructures have also been studied using CL to distinguish between polar and non-polar facet emissions [13]. While radial structures offer extensive active areas, axial MQW microrods—the focus of this work—provide unique advantages in terms of precise strain management along the growth direction and a more straightforward path for vertical carrier injection in device integration.

In addition to the geometric design, the fabrication methodology plays a decisive role in determining the optical and structural quality of AlGaN microrods. Generally, these architectures can be realized via either “bottom-up” epitaxial growth or “top-down” plasma etching. While the bottom-up approach avoids plasma-induced damage, it often suffers from significant inhomogeneity due to element segregation and non-uniform incorporation of aluminum at different crystallographic facets [14]. In contrast, the top-down approach employed in this work allows for superior control over the height and vertical alignment of the microrods while preserving the high crystalline quality and compositional uniformity of the original planar MQW epitaxial wafer.

However, the extension of these microrod architectures to high-Al-content AlGaN systems for UVC emission is comparatively underdeveloped. In such high-Al systems, the spatial distribution of carriers and the impact of surface states become even more critical. Specifically, the intricate relationships connecting microrod morphology, crystallographic facet evolution, size-dependent optical phenomena, and defect-related emission in the DUV regime have yet to be systematically elucidated.

In this study, we fabricated AlGaN microrods with diameters of 2, 3, and 4 μm using a top-down approach that combines inductively coupled plasma (ICP) dry etching with a subsequent wet chemical treatment. Through a correlative analysis using detailed morphological characterization, cathodoluminescence (CL) spectroscopy, and CL mapping, we systematically investigated the relationships between crystallographic facet formation, size-dependent emission properties (intensity and linewidth), and spatial emission distribution. This work clarifies the critical roles of size confinement and surface-related effects in modulating UVC spontaneous emission, providing experimental insights to guide the design of high-performance AlGaN-based deep-ultraviolet optoelectronic devices.

## 2. Materials and Methods

### 2.1. Sample Preparation

The samples used in this study were fabricated from a standard AlGaN-based UVC LED epitaxial wafer grown on a sapphire substrate grown by metal–organic chemical vapor deposition (MOCVD). The epitaxial structure consists of a 3 μm thick AlN layer, a 1.6 μm thick n-Al_0.62_Ga_0.38_N layer, a 0.6 μm thick n-Al_0.35_Ga_0.65_N layer, eight-period of Al_0.8_Ga_0.2_N (12 nm)/Al_0.62_Ga_0.38_N (3 nm) multiple quantum wells (MQWs), a 50 nm thick p-Al_0.88_Ga_0.12_N EBL and a 50 nm thick p-GaN layer. The estimated bandgap difference between the barrier and well layers is approximately 0.58 eV, corresponding to a conduction band offset (ΔE_C_) of ~0.41 eV and a valence band offset (ΔE_V_) of ~0.17 eV, indicating a Type-I band alignment.

**Figure 1:**
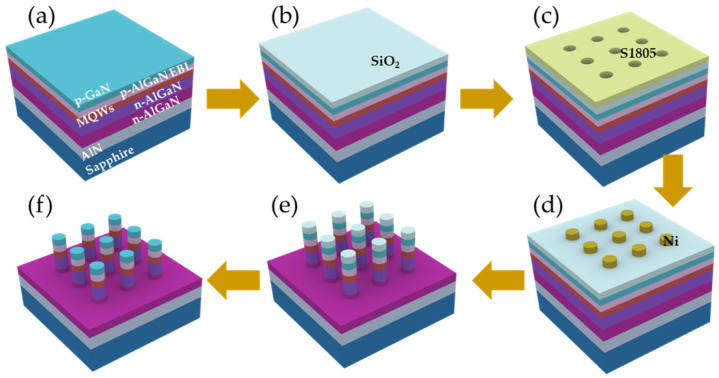
Fabrication process of the microrod cavities. The original epitaxial wafer (**a**), the SiO_2_ deposition (**b**), the Ni hard mask formed by photolithography and lift-off (**c**), etching of SiO_2_ (**d**), inductively coupled plasma itching of the wafer (**e**), and removal of the SiO_2_ (**f**).

The fabrication process of the AlGaN microrods is illustrated in Figure 1. Prior to patterning, the epitaxial wafer was immersed in diluted hydrochloric acid to remove surface oxides, followed by sequential ultrasonic cleaning in acetone, ethanol, and deionized water. A 500 nm thick SiO_2_ layer was then deposited by plasma-enhanced chemical vapor deposition (PECVD, Oxford Plasmalab 80plus, Oxford Instruments Plasma Technology, Bristol, UK). Positive photoresist (S1805) was spin-coated, and circular patterns with different diameters were defined by photolithography (Karl Suss MicroTec MA6/BA6, SUSS MicroTec, Garching, Germany). A 30 nm thick Ni film was deposited by electron beam evaporation (Kurt J. Lesker PVD 75, Kurt J. Lesker Company, Jefferson Hills, PA, USA) and subsequently lifted off in acetone to form circular Ni hard masks. Using the Ni disks as etching masks, the circular patterns were transferred into the SiO_2_ layer by inductively coupled plasma (ICP, Oxford Plasmalab System 100, Oxford Instruments Plasma Technology, Bristol, UK) etching. Subsequently, deep ICP etching was employed to transfer the defined patterns into the AlGaN epitaxial layers, forming microrods with well-controlled diameters. After dry etching, the remaining SiO_2_ layer was removed using buffered oxide etchant (BOE). To further eliminate etching-induced surface damage and improve sidewall quality, the samples were subjected to wet chemical treatment in a 2 mol/L KOH solution heated to 80 °C for 10 min. Through this combined dry and wet etching process, AlGaN microrods with diameters of 2 μm, 3 μm, and 4 μm were obtained.

### 2.2. Structural and Optical Characterization

High-resolution X-ray diffraction (HR-XRD) was employed to characterize the crystallographic orientations of the AlGaN microrods. The sidewall morphologies were examined using scanning electron microscopy (SEM, ZEISS GeminiSEM 360, Carl Zeiss AG, Oberkochen, Germany). Cathodoluminescence (CL, consisting of the GeminiSEM 360 integrated with a Horiba H-CLUE system) spectroscopy and CL mapping were performed to investigate the optical emission properties.

In this study, three different excitation power densities were achieved by varying the electron beam current while maintaining a constant accelerating voltage of 12 kV. Since the penetration depth of the electron beam depends solely on the accelerating voltage and the material composition, the interaction volume remains identical for all three power conditions. To quantitatively determine the probed depth and the corresponding sample layers, we employed two complementary models: the Kanaya–Okayama (K–O) range formula [15] and the Everhart–Hoff energy dissipation function [16]. The maximum electron penetration depth (the total range RR) was calculated using the Kanaya–Okayama equation, namely *R =* 0.026*AE*^1.67^*R*/*ρz*^0.89^ (μm), where *E* = 12 kV is the accelerating voltage, and A, ρ, and Z are the average atomic weight (g/mol), density (g/cm^3^), and average atomic number of each layer, respectively. For multilayer structures, the total range is obtained by summing the thicknesses of layers penetrated until the electron energy is fully dissipated, using the residual energy method. The relevant material parameters for each layer (p-GaN cap, p-AlGaN electron blocking layer, AlGaN multiple quantum wells, n-AlGaN buffer) were taken from the literature or calculated as weighted averages based on composition. The computation yields a total penetration depth of approximately R ≈ 0.85 μm at 12 kV, meaning the electron beam fully probes the p-GaN (0–50 nm) and p-AlGaN EBL (50–100 nm), completely traverses the MQW stack (100–220 nm), and partially penetrates the underlying n-AlGaN buffer layer (220 nm to ~0.85 μm).

To assess where the majority of electron–hole pairs are generated, we used the Everhart–Hoff energy dissipation model, which describes the normalized depth distribution of energy deposition. The normalized depth is defined as *y* = *z*/*R*, where z is the depth from the surface. The energy dissipation function λ(*y*) = 0.60 + 6.21*y* – 12.40*y*^2^ + 5.69*y*^3^ exhibits a maximum at y_max_ ≈ 0.3. Thus, the depth of peak carrier generation is z_max_ = y_max_ × R ≈ 0.255 μm. Although this peak lies slightly below the MQW region, the entire MQW stack lies on the rising flank of the dissipation curve, where the energy deposition is still intense. Consequently, the MQWs are subjected to high carrier generation rates, and the high excitation density at 12 kV provides sufficient photon flux to overcome the strong UV self-absorption by the very thin p-GaN cap layer.

The excitation mode was point excitation, with an electron beam spot size of approximately 20 nm and an integration time of 2 s. Multiple microrods with different diameters were measured under identical conditions to ensure reliable comparison. Spatially resolved CL mapping was further carried out to obtain two-dimensional emission distributions, local spectra, and quantitative parameters, including emission intensity, peak position, and full width at half maximum (FWHM). The working distance was set to 9.3 mm to achieve sufficient spatial resolution for resolving emission variations across the microrods.

## 3. Results and Discussion

### 3.1. Morphological Analysis

To determine the crystallographic orientations of the sidewalls and etching pits in the AlGaN microrods, X-ray diffraction (XRD) *φ*-scan measurements of the r-plane were performed. Within a scanning range of 180°, diffraction peaks appeared at 60° intervals, originating from the sixfold symmetry of the wurtzite crystal structure. By rotating the sample to one diffraction peak position, the horizontal cross-sectional direction was aligned with the m-plane, followed by side-view observations using scanning electron microscopy (SEM).

**Figure 2:**
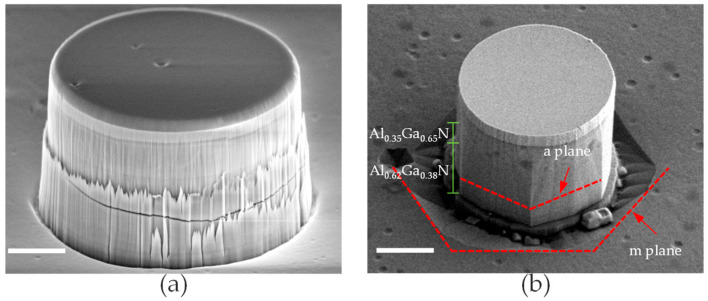
SEM side-view images of AlGaN microrod (**a**) before and (**b**) after modification. The scale bars represent 2 μm.

Figure 2 shows the side-view SEM images of the AlGaN microrods. Before KOH treatment, the microrod is a lithographically defined cylinder, and the sidewalls show roughness caused by ICP etching, as shown in Figure 2a. After treatment with KOH, the microrod exhibited a prismatic structure of specific crystal planes as shown in Figure 2b. In the high-Al-content AlGaN region of the microrod, the sidewalls form an angle of approximately 30° with respect to the m-plane direction, while the bottom facets of the etching pits remain parallel to the m-plane. Based on the geometrical relationships among crystallographic planes in the wurtzite structure, the sidewalls are identified as a-planes, whereas the bottom facets correspond to m-planes.

Figure 2b show that following KOH treatment, the sidewalls become smoother and evolve into stable facets dominated by a-planes, while the etching pits expand outward and stabilize as m-plane-related semi-polar facets. After KOH treatment, it is very unusual for high Al-content components to spontaneously exhibit the a-plane. In numerous past studies, it has been found that for GaN-based materials, the crystal plane that spontaneously appears after KOH treatment is always the m-plane [17,18,19]. Therefore, AlN and materials with high Al content have crystal plane characteristics different from those of GaN. The top part of the microrod is made of low-Al-content AlGaN and GaN, so it still tends to form m-planes. However, because the thickness of this part is too small, it ultimately appears nearly circular.

This facet-selective etching behavior can be attributed to differences in atomic configurations between non-polar planes. For AlN, the surface atomic density of the a-plane (0.745 Å^−2^) is higher than that of the m-plane (0.645 Å^−2^), resulting in greater chemical stability of the a-plane [20]. In alkaline KOH solution, negatively charged OH^−^ ions experience stronger electrostatic repulsion near the a-plane family, whereas the repulsion is weaker near the m-plane family. As a result, m-plane-related facets are preferentially etched during wet chemical treatment, while a-plane facets tend to be preserved. Consequently, the microrod sidewalls are dominated by a-plane facets with minor residual ridges originating from m-plane segments, whereas the bottom etching pits preferentially expose m-plane-related facets during outward etching.

The observed facet evolution behavior is consistent with previous reports on top-down fabricated GaN and AlN micro- and nanostructures [21,22,23,24]. Similar facet selectivity, featuring a-plane or a-plane-related semi-polar sidewalls and m-plane-dominated bottom facets, has been reported in high-aspect-ratio GaN/AlN nanopillars fabricated by combined dry and wet etching processes [21,22,23]. In addition, ultrasharp periodic AlN nanotips fabricated by thinning processes also exhibit a-plane-related semi-polar sidewalls with an angle of approximately 30° relative to the m-plane at the bottom facets [24]. These results indicate that facet separation toward a-plane and m-plane families during wet chemical etching is highly dependent on the Al component in the wurtzite III-nitride materials.

**Figure 3:**
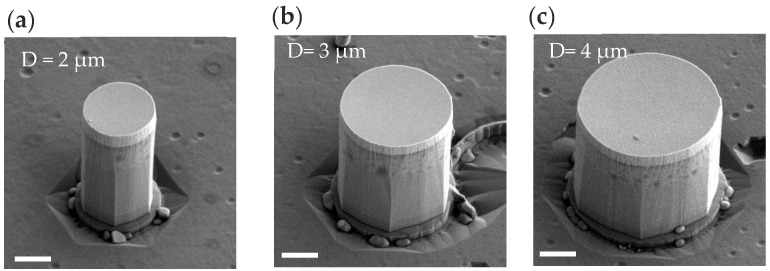
SEM side-view images of microrods with different sizes. (**a**) 2 μm, (**b**) 3 μm, (**c**) 4 μm. The scale bars represent 1 μm.

Figure 3 presents SEM images of microrods with diameters (D) of 2 μm, 3 μm, and 4 μm, and exhibit consistent facet characteristics. The lower half of the high-alumina component in all microrods exhibits a clear a-face, while the crystal face features of the upper half of the low-alumina component gradually disappear.

Overall, the microrod morphology is governed by both etching conditions and intrinsic crystallographic properties. The formation of a-plane sidewalls provides a well-defined structure for the size-dependent optical properties discussed below.

### 3.2. Point Spectral Analysis

**Figure 4:**
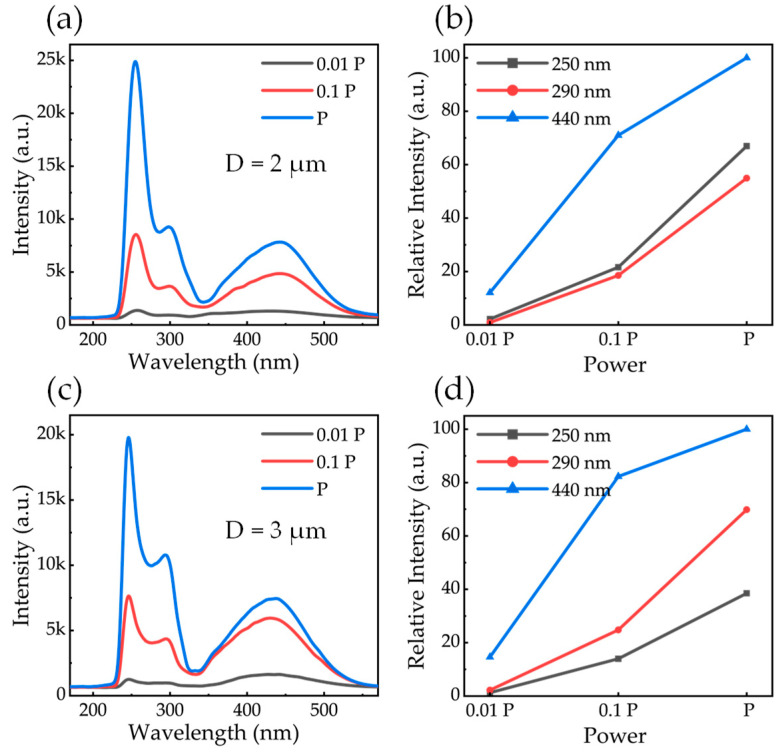

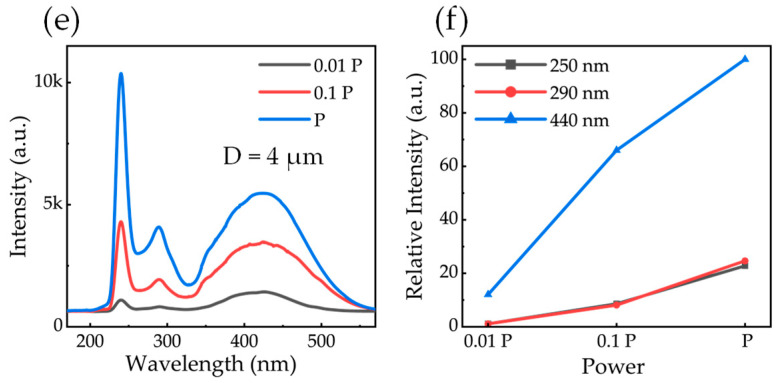
CL from (**a**) 2 μm, (**c**) 3 μm, (**e**) 4 μm microrod. The dependence of integrated emission intensity on excitation power is shown in (**b**,**d**,**f**) for each emission peak. Error bars derived from the fitting uncertainties are present for all data points but are largely obscured by the symbols due to their small values.

The size-dependent optical properties were investigated by performing point-excitation CL spectra from individual AlGaN microrods with diameters of 2, 3, and 4 μm, as presented in Figure 4. To elucidate the underlying recombination dynamics, spectra were recorded at three distinct excitation currents, 5.0, 0.5, and 0.05 nA, corresponding to relative power densities of P, 0.1P, and 0.01P, respectively, while maintaining identical collection conditions.

The representative spectra in Figure 4a,c,e consistently reveal three distinct emission peaks for all microrods. The dominant feature is a DUV emission centered at ~250 nm, which is accompanied by a shoulder-like feature at ~290 nm and a broad blue luminescence band near 440 nm. Crucially, the evolution of these bands with increasing excitation power is markedly different, indicating that they come from different recombination.

**Figure 5:**
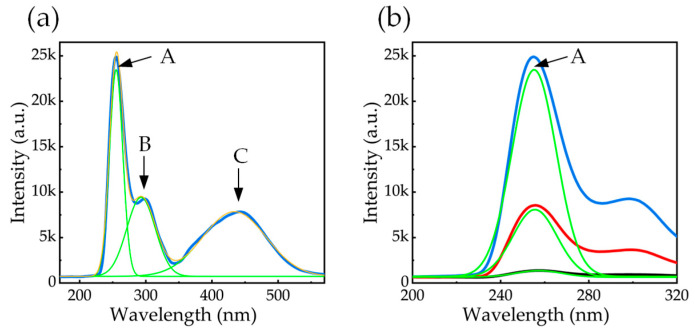
Gaussian fitting of the CL spectrum of 2 μm diameter AlGaN microrod (**a**) and band-edge emission peak (**b**).

Figure 4b,d,f summarizes the integrated emission intensities as a function of excitation power for different microrod diameters. To ensure accuracy, these intensities were determined by deconvolving the CL spectra into individual Gaussian peaks (as shown in Figure 5). Note that error bars, representing the uncertainties derived from the Gaussian fitting of each individual spectrum, are included for all data points; however, they are smaller than the symbol size and thus largely obscured. The 250 nm and 290 nm emissions, despite having lower integrated areas compared to the defect band, display a robust superlinear dependence on excitation power. Specifically, the 250 nm emission intensity increases significantly with decreasing microrod diameter, representing the band-edge recombination within the MQWs. The 290 nm peak, assigned to recombination in the lower-Al-content AlGaN layer, follows a similar size-dependent trend.

In contrast, while the broad 440 nm emission exhibits the highest total integrated intensity across all samples, its growth tends toward saturation at higher excitation levels—a behavior characteristic of deep-level defect recombination. In AlGaN, this blue luminescence is widely attributed to carbon-related defects (such as C-N), which serve as radiative recombination centers with a limited density of states [25,26]. The behavior between the band-edge and defect emissions reflects the competition for carriers, where the high-efficiency MQW channel increasingly dominates as the defect states become occupied.

To deconvolve the spectral components, the CL spectrum of a representative microrod was fitted using a multi-peak Gaussian function (Figure 5). The analysis yields three distinct peaks, labeled A, B, and C. These peaks are assigned to band-edge recombination in the MQWs (A), the lower-Al-content AlGaN layer (B), and carbon-related deep-level defects (C), respectively. The extracted center wavelengths for each peak are consistent with these physical assignments.

**Figure 6:**
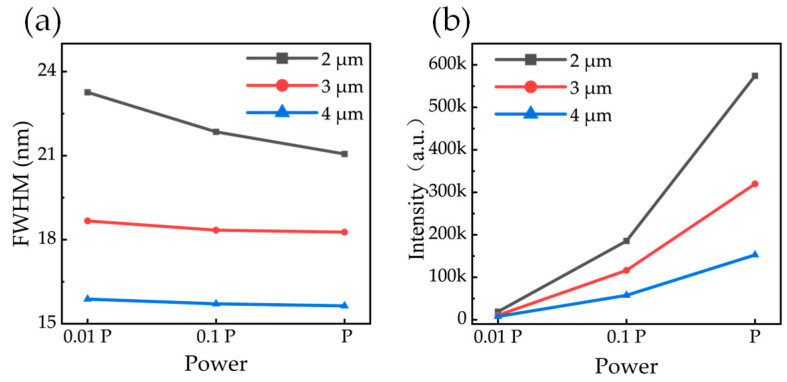
Variation in FWHM for samples of different sizes (**a**) and variation in the peak intensity of the band-edge emission (**b**).

The evolution of the band-edge emission FWHM with excitation power reveals distinct differences in carrier recombination dynamics between the microrods (Figure 6a). The 4 µm microrod displays a consistently narrow FWHM, indicating a high degree of material homogeneity. Conversely, the 2 µm microrod exhibits a significantly broader FWHM at low excitation, which progressively narrows at higher powers.

This behavior is governed by the interplay between carrier diffusion and size-dependent strain relaxation, which becomes increasingly prominent in structures with a high surface-to-volume ratio. Although the electron interaction volume at 12 kV is localized at the microrod center, carrier diffusion allows a significant fraction of electron–hole pairs to migrate toward the sidewalls. These surface states create a broad distribution of energy levels that act as alternative recombination pathways. Furthermore, the top-down etching process induces global strain relaxation throughout the entire microrod volume. In smaller structures, this more complete release of epitaxial strain modulates the internal polarization fields and band-edge potential fluctuations.

At low carrier densities, recombination occurs across this complex energy landscape influenced by both surface states and residual strain, resulting in a marginal spectral broadening. However, under high excitation, the system undergoes a state-filling effect: the more efficient, intrinsic band-edge recombination channel becomes saturated with carriers and dominates the emission spectrum. This saturation diminishes the relative contribution of the broader surface-state recombination and the impact of potential fluctuations, leading to the observed line-narrowing in the 2 μm microrod.

A strong enhancement in band-edge emission intensity is also observed as the microrod diameter decreases (Figure 6b). Under identical excitation conditions, the integrated intensity from the 2 µm microrods is 2.76 times greater than that from the 4 µm structures. This intensity boost is a key signature of the Purcell effect, where the optical cavity formed by the microrod increases the spontaneous emission rate. By accelerating radiative recombination, the cavity allows emission to more effectively compete with non-radiative processes, thereby boosting the overall light output. The previously discussed line-narrowing behavior further supports this conclusion, as the Purcell effect selectively enhances the primary band-edge recombination channel that comes to dominate the spectrum at high power [27].

### 3.3. Spatially Resolved Spectral Analysis

**Figure 7:**
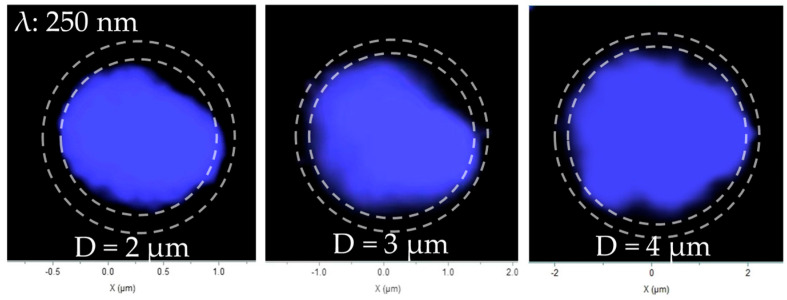
CL mapping images of samples with different sizes. Outer dashed lines indicate the microrod edges; inner dashed lines indicate the boundaries of the band-edge emission regions.

To directly visualize the impact of surface recombination, we performed spatially resolved cathodoluminescence (CL) mapping at an acceleration voltage of 12 kV and a beam current of 5 nA, ensuring sufficient excitation of the active MQW region. The monochromatic maps, taken at the 250 nm band-edge emission, reveal a luminescent core surrounded by a non-radiative “dead layer” at the microrod perimeter (Figure 7). The dead layer length (L) was defined as the difference between the physical radius (R) and the effective luminescent radius (R_lum_), expressed as L = R − R_lum_. Here, R was measured from SEM images, while R_lum_ was extracted from the intensity profiles of the CL maps. This dead layer represents the region where charge carriers are depleted by rapid non-radiative recombination at the surface states.

**Figure 8:**
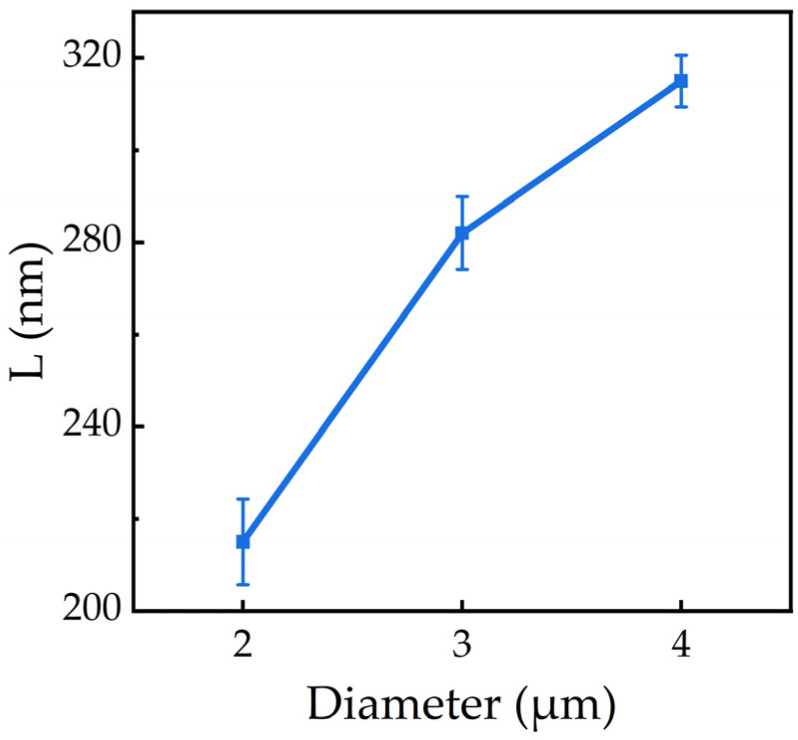
Variation in the non–band-edge emission width for microrod of different sizes.

Furthermore, the width of this dead layer exhibits a strong dependence on the microrod diameter. As summarized in Figure 8, the dead layer width increases with diameter before beginning to saturate. This trend is governed by carrier diffusion: in larger microrods, carriers generated deeper within the core can diffuse a greater distance before reaching the surface, resulting in a wider apparent region of suppressed emission near the edge.

For the spatial analysis of CL images, all quantitative values, including the luminescent area and dead layer width, were averaged over three randomly selected microrods of the same dimensions to ensure reproducibility. The corresponding uncertainties are reported as standard deviations in the respective plots.

This size-dependent behavior is governed by a competition between two key processes: radiative recombination within the quantum wells and non-radiative recombination at the etched sidewalls. In smaller microrods, the small diameter provides stronger strain relaxation. This reduces band bending near the microrod edge, suppressing electron diffusion toward the sidewalls [28] and the quantum-confined Stark effect (QCSE), and leading to greater electron–hole wavefunction overlap and a higher radiative recombination rate. As a result, carriers recombine radiatively before they can diffuse to the surface, resulting in a narrower non-emissive “dead layer.”

Conversely, in larger microrods, strain relaxation is less complete. The stronger QCSE suppresses radiative efficiency, increasing the carrier lifetime. This provides more time for carriers to diffuse laterally toward the sidewalls, where they are captured by etch-induced defect states and recombine non-radiatively. The dead layer width, therefore, increases with diameter. However, this width is ultimately limited by the carrier diffusion length, explaining the slower increase observed for the largest microrods (Figure 8).

**Figure 9:**
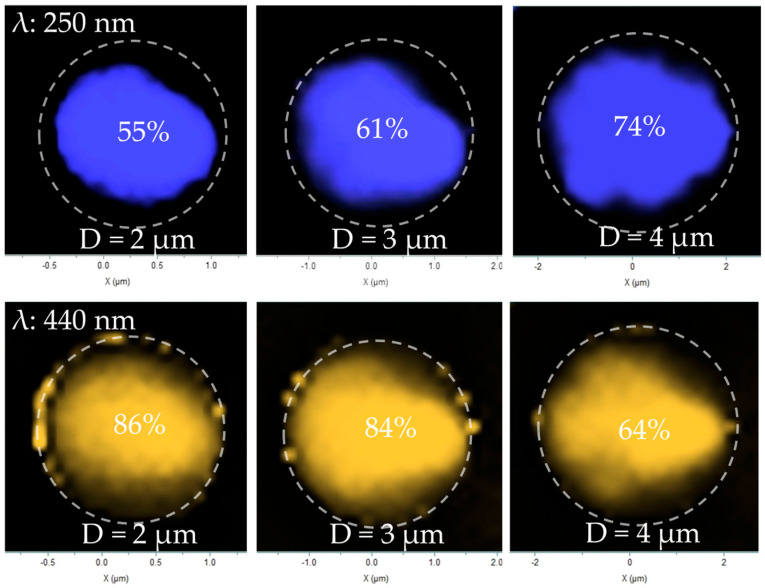
CL mapping images of different luminescence peaks. Dashed lines indicate the microrod edge contours.

To identify the source of the non-radiative regions, we compared the spatial distribution of the band-edge emission with that of the defect-related luminescence (Figure 9). In this analysis, the effective luminescent area was determined through image processing by integrating the total number of pixels in the “bright” statistical region. The maps reveal a clear anti-correlation: the bright band-edge emission is concentrated in the microrod core, while the defect emission is also dominant at the perimeter, spatially coinciding with the non-emissive “dead layer.”

**Figure 10:**
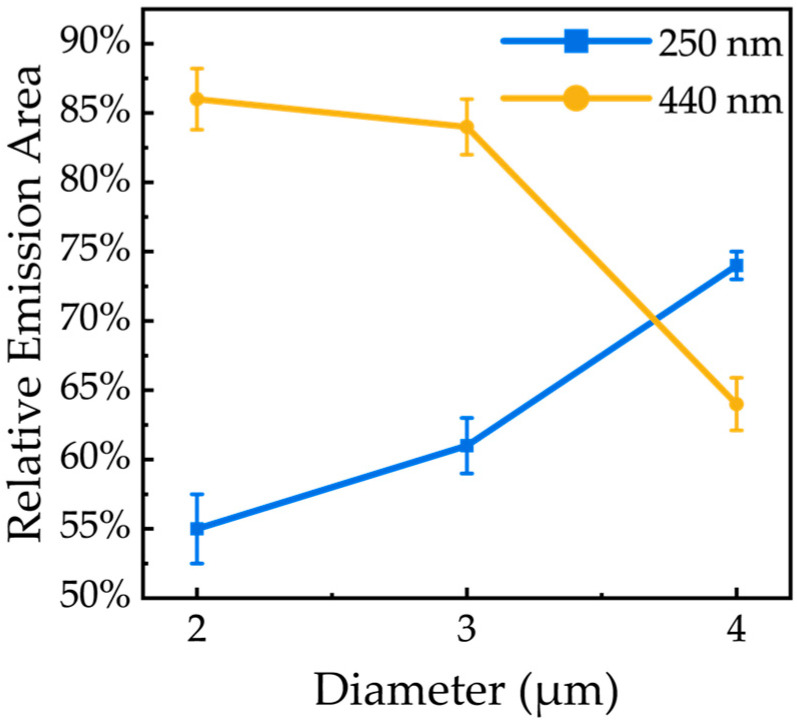
Relative emission areas of different luminescence peaks.

This spatial competition is quantified in Figure 10. As the microrod diameter increases from 2 µm to 4 µm, the fractional area of band-edge emission grows significantly from 55% to 74%. Concurrently, the area dominated by defect-related emission shrinks from 86% to 64%. At this point, an interesting pattern can be observed: although the band-edge emission volume ratio in small-sized microrods is smaller than that in large-sized microrods, the overall band-edge emission intensity is stronger (as seen in Figure 6b), which demonstrates a change in the radiative mechanism in the small-sized microrods.

Overall, the 2 µm AlGaN microrods emerge as the optimal structure, exhibiting superior band-edge emission intensity, excitation-induced linewidth narrowing, and distinct spatial emission. This peak performance arises from a synergy between two size-dependent effects. First, like other smaller microrods, they benefit from significant strain relaxation, which suppresses the QCSE and enhances intrinsic radiative recombination. Crucially, however, it is at the 2 µm diameter that the Purcell effect becomes most pronounced, further accelerating spontaneous emission. This combined enhancement of both radiative efficiency and emission rate defines 2 µm as a critical dimension for tailoring the optical properties of AlGaN microrod structures.

## 4. Conclusions

In summary, the facet evolution and size-dependent emission behavior of AlGaN microrods fabricated by dry etching followed by KOH wet chemical modification were investigated. We found that the KOH treatment preferentially forms a-plane-dominated sidewalls on the high-Al-content regions of the microrods, whereas the etch pit bottoms stabilize as m-plane facets. Optical characterization reveals smaller microrods exhibiting enhanced band-edge emission and excitation-dependent linewidth narrowing. In particular, the band-edge emission intensity of the 2 μm microrods is enhanced by a factor of 3.76 compared to the 4 μm structures. CL mapping further unveils the competitive dynamics between radiative recombination within the quantum wells and non-radiative recombination at surface states. These results identify 2 μm as the most favorable dimension among the studied samples for maximizing spontaneous emission in AlGaN microrods and emphasize the importance of facet engineering and size confinement in designing high-performance deep-ultraviolet optoelectronic devices.

## Data Availability

All data, theory, details that support the findings of this study are available from the corresponding authors upon reasonable request.
